# FASN thioesterase domain mutation and fatty acid profile in Holstein Friesian intramuscular fat

**DOI:** 10.5194/aab-68-271-2025

**Published:** 2025-05-08

**Authors:** David Cancino-Baier, Erwin Muñoz-Acuña, John Quiñones, Rommy Diaz, Jorge F. Beltrán, Jorge Farías, Karla Inostroza, Rodrigo Huaiquipan, José Bento Sterman Ferraz, Néstor Sepúlveda

**Affiliations:** 1 Facultad de Ciencias Agropecuarias y Medioambiente, Universidad de La Frontera, 01145 Francisco Salazar Ave, Temuco, Chile; 2 Centro de Excelencia en Física e Ingeniería en Salud, Facultad de Ingeniería y Ciencias, Universidad de La Frontera, 01145 Francisco Salazar Ave, Temuco, Chile; 3 Programa de Doctorado en Ciencias Mención Biología Celular y Molecular Aplicada, Universidad de La Frontera, 01145 Francisco Salazar Ave, Temuco, Chile; 4 Centro de Excelencia en Biotecnología de la Reproducción (CEBIOR-BIOREN), Universidad de La Frontera, 01145 Francisco Salazar Ave, Temuco, Chile; 5 Departamento de Ingeniería Química, Facultad de Ingeniería y Ciencias, Universidad de La Frontera, 01145 Francisco Salazar Ave, Temuco, Chile; 6 Departamento de Ciencias Agropecuarias y Acuícolas, Facultad de Recursos Naturales, Universidad Católica de Temuco, Temuco 4780000, Chile; 7 Programa de Doctorado en Ciencias Agropecuarias y Medioambiente, Universidad de La Frontera, 01145 Francisco Salazar Ave, Temuco, Chile; 8 Grupo de Melhoramento Animal, Departamento de Ciencias Baìsicas, Faculdade de Zootecnia e Engenharia de Alimentos, Universidade de São Paulo, Pirassununga, SP, Brazil; 9 Departamento de Producción Agropecuaria. Facultad de Ciencias Agropecuarias y Medioambiente, Universidad de La Frontera, 01145 Francisco Salazar Ave, Temuco, Chile

## Abstract

A mutation in the thioesterase domain of the FASN enzyme is associated with a lower percentage of myristic acid when the mutated allele is present, although the underlying mechanism remains unclear. The objective of this study was to evaluate, through in silico simulation, the effect of this mutation on the affinity of the catalytic site of the thioesterase domain for two of its ligands, myristoyl and palmitoyl, and its impact on the fatty acid profile of intramuscular fat. Animals were genotyped using PCR–RFLP, and fatty acid composition was assessed by gas chromatography. Simulations were conducted using the Bos taurus reference sequences (Q71SP7) from the UniProt database. Tertiary structures were generated through homology modeling using PHYRE2. The resulting models were refined with GalaxyRefine and evaluated by generating Ramachandran plots in SWISS-MODEL. Molecular docking was performed with AutoDock Vina. Four molecular dockings were conducted between both enzyme models, wildtype and mutated, and the ligands, resulting in the following combinations: wildtype/palmitoyl, mutated/palmitoyl, wildtype/myristoyl, and mutated/myristoyl. The interaction regions of the generated dockings were visualized using PyMOL software. A decrease in the percentage of myristic and palmitic fatty acids was observed in homozygous individuals with the mutated allele. The mutated/myristoyl complex showed a higher interaction compared to the wildtype/myristoyl complex. However, despite the increased affinity between the mutated enzyme and myristoyl, the possible alteration in the enzyme's structure is likely more relevant in affecting the fatty acid profile of intramuscular fat.

## 1 Introduction

Meat is one of the most important nutritional sources globally (Xie et al., 2022), with beef being one of the most widely consumed meat products (Whitton et al., 2021). However, the shift towards healthier lifestyles has driven the demand for meat products that are not only nutritious but also promote overall health (Teixeira and Rodrigues, 2021). For instance, in Switzerland, students have expressed concerns about meat consumption (Arnaudova et al., 2022), and in Finland young adults have shown a reduction in meat intake (Knaapila et al., 2022). Meanwhile, in the Netherlands, meat consumption has remained stable (Dagevos and Verbeke, 2022), whereas in Spain consumption is high despite recommendations to reduce it for health reasons (Font-i-Furnols and Guerrero, 2022). In contrast, Uruguay maintains a strong culture of meat consumption (Realini et al., 2022). The composition of fatty acids (FAs) in meat has intrigued scientists due to the correlation between high meat consumption and increased mortality risk, which is attributed to the high content of saturated FAs, cholesterol, and iron (Wang et al., 2022). Research on improving the quality of meat-containing foods aims to reduce saturated fat consumption, thereby enhancing their overall health benefits (López-Pedrouso et al., 2021). While several studies suggest a decline in meat consumption (Graça et al., 2019), the importance of beef should not be underestimated, as it provides essential nutrients necessary for bodily function and development, such as high-quality proteins, iron, and vitamins, making it a significant component of the human diet (Leroy et al., 2022; De Smet and Vossen, 2016).

The enzyme fatty acid synthase (FASN) is recognized as the primary synthesizer of fatty acids, particularly long-chain FAs like myristic and palmitic acids. A mutation in this enzyme (SNP FASN g.17924A > G) has been associated with a reduction in the content of myristic acid (C14:0) when the mutated allele is present (Maharani et al., 2012). This mutation is a single-nucleotide polymorphism located within the thioesterase (TE) domain at position 2264 of the amino acid sequence, resulting in the substitution of threonine with alanine (Cancino-Baier et al., 2021). The TE domain is linked to the acyl carrier protein, which transports the emerging FA across the various catalytic sites of the enzyme. Once the chain reaches a length of 16 carbons (C), the TE domain recognizes the palmitoyl ACP and induces the release of the FA (Chakravarty et al., 2004). While the ideal length for the released fatty acid is 16 C, the TE domain can also recognize and release FAs with chain lengths both above 18 and below 16 C (Mattick et al., 1983; Pazirandeh et al., 1989). Myristic and palmitic fatty acids play significant biological roles in lipid metabolism and fat synthesis in animals, particularly in cattle. Myristic acid, which is present in smaller amounts compared to other fatty acids in intramuscular fat, is converted into myristoyl CoA and participates in triglyceride synthesis (Hu et al., 2023). On the other hand, palmitic acid, one of the most abundant fatty acids in intramuscular fat, along with stearic acid, constitutes the majority of the saturated fatty acids present in this fat (Zhang et al., 2015). These saturated fatty acids are important in the composition of intramuscular fat and can influence its nutritional and organoleptic properties.

Therefore, the aim of this study was to assess, through in silico simulation, the effect of this mutation on the affinity of the catalytic site of the TE domain for two of its ligands, myristoyl and palmitoyl, and its impact on the composition of FAs in intramuscular fat.

## 2 Materials and methods

This study followed the animal care protocol approved by the Scientific Ethical Committee of Universidad de La Frontera, Temuco, Chile (Protocol no. 033-17). *Longissimus* muscle samples were collected from 196 Holstein Friesian steers slaughtered at Planta Faenadora de Carnes Victoria S.A. located in Victoria, Araucanía region, Chile (lat: - 38.2281; long: - 72.3331; alt: 352 m a.s.l.). The samples were rapidly frozen in liquid nitrogen and stored at - 80 °C until DNA and total FA extractions were conducted. All steers were 18 months old and were raised under identical conditions at the local dairy farm Agrícola Ancali in Los Angeles, Bio-Bio region, Chile.

### 2.1 Extraction of DNA and genotyping of single-nucleotide polymorphisms (SNPs)

Genomic DNA was extracted from animal muscle tissue using a commercial kit (Cat. #D3024, Quick-DNA Miniprep kit, Zymo Research, California, USA). Genotyping for the FASN g.17924A > G SNP was performed using the PCR–RFLP technique. The PCR, with a total volume of 50 µ L, included 1X PCR buffer, 0.8 mM dNTPs, forward primer (5'-AGAGCTGACGGACTCCACAC-3') and reverse primer (5'-CTGCATGAAGAAGCACATGG-3') (10 ng of each primer), Paq5000 polymerase (2.5 U), 37 µ L of nuclease-free water, and 50 ng of genomic DNA. The PCR was conducted following the Paq5000 DNA polymerase protocol (Cat. #600680, Agilent Technologies, California, USA).

A 759 bp amplicon was generated from this reaction, which was subsequently digested using the MscI restriction enzyme (Cat. #R0534S, New England Biolabs, Massachusetts, USA) via the RFLP technique. The RFLP reaction mixture consisted of 3.1 µ L of 1X CutSmart buffer, 0.5 µ L of the MscI restriction enzyme (3 U), 6.4 µ L of nuclease-free water, and 10 µ L of PCR product (10 ng µ L^−1^), yielding a total volume of 20 µ L. The reaction was incubated at 37 °C overnight. The RFLP products were analyzed by agarose gel electrophoresis (1.5 %). The AA genotype produced three fragments of 342, 251, and 166 bp. The AG genotype produced four fragments of 417, 342, 251, and 166 bp. The GG genotype produced two fragments of 417 and 342 bp.

### 2.2 Composition of fatty acids in intramuscular fat

Total lipids were extracted from the *Longissimus* muscle (intramuscular adipose tissue) following the method described by Folch et al. (1957). FAs were subjected to cold methylation in the presence of KOH, methanol, and hexane, as outlined by Inostroza et al. (2012). The resulting FA methyl esters were analyzed using a Clarus 500 gas chromatograph (Perkin Elmer, USA) equipped with an autosampler and an SPTM Fused Silica Capillary Column 2380 (60 m × 0.25 mm × 0.2 µ m film thickness, Supelco, USA) and coupled to a flame ionization detector (FID, Perkin Elmer, USA). Nitrogen was used as the carrier gas at a flow rate of 45 mL min^−1^ (Inostroza et al., 2012). The temperature program was as follows: initial pre-heating at 150 °C for 1 min, followed by a temperature increase of 1 °C min^−1^ up to 168 °C (maintained for 11 min) and a final increase of 6 °C min^−1^ up to 230 °C (maintained for 8 min), resulting in a total cycle time of 48.3 min (Inostroza et al., 2012).

### 2.3 In silico evaluation of the enzyme interaction with ligands, myristoyl and palmitoyl

The Bos taurus reference sequences, identified by the code Q71SP7 and associated with the thioesterase domain of the FASN enzyme, were downloaded from the UniProt database (The UniProt, 2018) and used for this evaluation. An in silico mutation was introduced in this sequence, corresponding to the substitution of threonine (THR) with alanine (ALA) at position 2264 (Cancino-Baier et al., 2021), resulting in an alternative sequence (FASN^−^).

Homology modeling was performed using the PHYRE2 program (Kelley et al., 2015) to generate tertiary structures from the sequences mentioned above (FASN and FASN^−^). The resulting models were then refined using the GalaxyRefine tool (Heo et al., 2013). The quality of these models was subsequently evaluated by generating Ramachandran plots using the SWISS-MODEL platform (Waterhouse et al., 2018).

Molecular docking was conducted using the AutoDock Vina program (Trott and Olson, 2009) integrated into the PyRx software (Dallakyan and Olson, 2015). Docking simulations were performed on the homology-modeled structures (FASN and FASN^−^), with the catalytic site identified by the following amino acids: ASN-16, LEU-17, VAL-18, ILE-44, GLU-45, PHE^−164^, PHE^−165^, PHE^−212^, ALA^−213^, SER^−216^, PHE^−217^, and LYS^−220^ (Pazirandeh et al., 1989). The ligands used in this study, myristoyl and palmitoyl, were obtained from the ChemSpider database (Pence and Williams, 2010), with reference codes ID:10140118 and ID:559149, respectively.

Four molecular docking simulations were carried out between the enzyme models and the ligands as follows: FASN/palmitoyl (parameters: center x=6.7964 ; center y=2.9599 ; center z=33.0859 ; size x=25 ; size y=25 ; size z=25 ; and exhaustiveness = 8.0), FASN^−^/palmitoyl (parameters: center x=7.2681 ; center y=3.4814 ; center z=31.2852 ; size x=25 ; size y=25 ; size z=25 ; and exhaustiveness = 8.0), FASN/myristoyl (parameters: center x=6.7964 ; center y=2.9599 ; center z=33.0859 ; size x=25 ; size y=25 ; size z=25 ; and exhaustiveness = 8.0), and FASN^−^/myristoyl (parameters: center x=7.2681 ; center y=3.4814 ; center z=31.2852 ; size x=25 ; size y=25 ; size z=25 ; and exhaustiveness = 8.0). The interaction regions of the resulting docking complexes were visualized using PyMOL software (PyMOL Molecular Graphics System, Version 2.3.1, Schrödinger, LLC).

The averages and standard deviations were calculated by repeating each docking simulation 10 times, using AutoDock Vina (Trott and Olson, 2009) and PyRx (Dallakyan and Olson, 2015).

### 2.4 Analysis of statistical data

Molecular docking results were analyzed using ANOVA with the SPSS v.23 statistical package (IBM, USA), based on the obtained affinity values. A 95 % confidence interval ( p=0.05 ) was applied, followed by a Tukey post hoc test. The same analysis was conducted to evaluate the fatty acid profile across the genotypes.

## 3 Results

### 3.1 Fatty acid profile of intramuscular fat

The presence of the SNP FASN g.17924A > G significantly influenced the intramuscular fat profile, where a reduction in the proportions of myristic (C14:0) and palmitic (C16:0) FAs was observed in individuals with the homozygous mutated genotype (Table 1). A slight increase in saturated FAs (SFAs) and a decrease in monounsaturated FAs (MUFAs) were also noted when comparing the AG and GG genotypes, while polyunsaturated FAs (PUFAs) showed no statistically significant differences between the three genotypes. A notable difference between the groups was observed in omega-3 (n3) FAs, with the heterozygous genotype exhibiting the highest percentage. Consequently, the omega-6 / omega-3 ratio in this tissue was affected, with the heterozygous genotype displaying a lower ratio of these FAs.

**Table 1:** Percentage of fatty acids in intramuscular fat according to genotype (means ± standard error).

	FASN g.17924A > G SNP genotypes			
Fatty acids	AA ( n=20 )	AG ( n=20 )	GG ( n=20 )	p value
C14:0	3.43 ± 0.09^a^	3.30 ± 0.64^a^	3.05 ± 0.04^b^	0.001
C16:0	27.92 ± 0.32^a^	27.27 ± 0.24^a^	26.43 ± 0.09^b^	0.001
C18:0	20.34 ± 0.57	21.30 ± 0.26	21.53 ± 0.42	0.274
OLEICO	40.52 ± 0.43	39.58 ± 0.47	39.79 ± 0.46	0.629
SFAs	52.69 ± 0.64^a,b^	53.83 ± 0.26^a^	51.16 ± 0.54^b^	0.000
MUFAs	44.34 ± 0.56^a,b^	42.76 ± 0.41^a^	45.18 ± 0.55^b^	0.001
PUFAs	2.88 ± 0.16	3.01 ± 0.12	3.16 ± 0.06	0.437
n3	0.29 ± 0.01^a^	0.35 ± 0.01^b^	0.28 ± 0.00^a^	0.000
n6	2.80 ± 0.21	2.61 ± 0.13	2.82 ± 0.06	0.418
PUFAs : SFAs	0.06 ± 0.00	0.05 ± 0.00	0.06 ± 0.00	0.434
n6 : n3	9.92 ± 0.69^a^	8.17 ± 0.40^a,b^	9.77 ± 0.20^a,c^	0.003

^a,b,c^ Different letters in the same row indicate a statistical difference ( p<0.05 ). SFAs: saturated fatty acids. MUFAs: monounsaturated fatty acids. PUFAs: polyunsaturated fatty acids.

### 3.2 In silico evaluation of FASN's interaction with its ligands, myristoyl and palmitoyl

**Figure 1:**
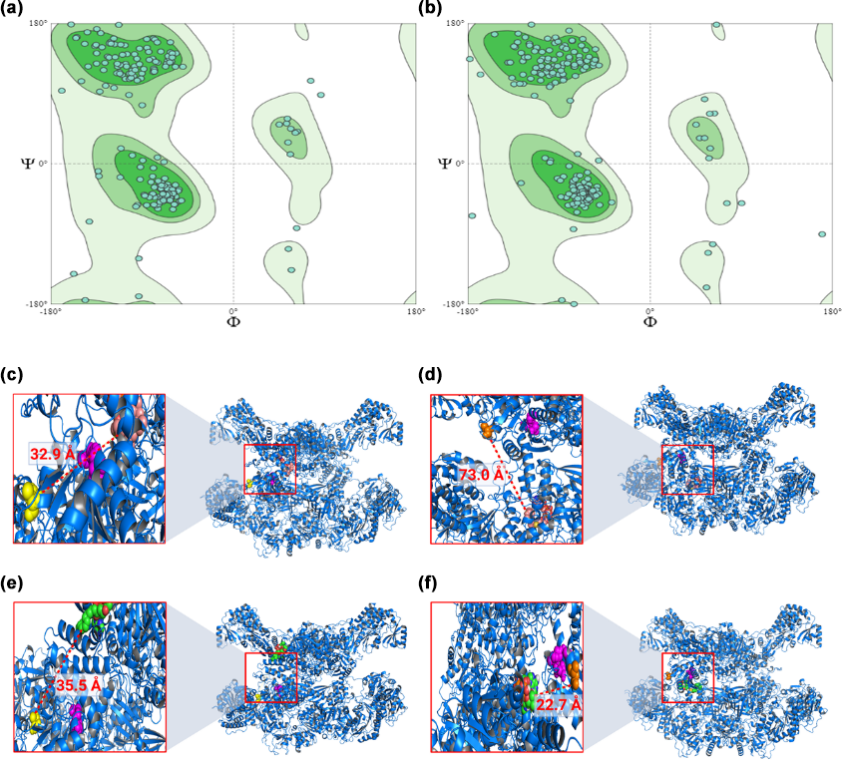
Ramachandran graphs for the models generated from FASN **(a)** and FASN^−^ **(b)**, together with molecular links between FASN and its ligands: FASN/palmitoyl **(c)**, FASN^−^/palmitoyl **(d)**, FASN/myristoyl **(e)**, and FASN^−^/myristoyl **(f)**. In the zoom you can see the catalytic pocket in detail. In pink the catalytic site is highlighted, in yellow the threonine, and in orange the alanine. In dark pink the palmitoyl is indicated and in green the myristoyl. The distance between the modified amino acid and the substrate, in Ångström (Å), is also indicated.

The models obtained through homology modeling demonstrated good quality, as indicated by the Ramachandran plots, with a high percentage of residues located in favorable regions: 95.71 % for FASN and 93.07 % for FASN^−^ (Fig. 1a and b). In contrast, statistical analysis of the molecular docking results revealed that the FASN/myristoyl complex exhibited lower interaction due to a significant decrease in affinity ( p=0.0115 ), whereas no significant differences were observed in the other complexes.

**Figure 2:**
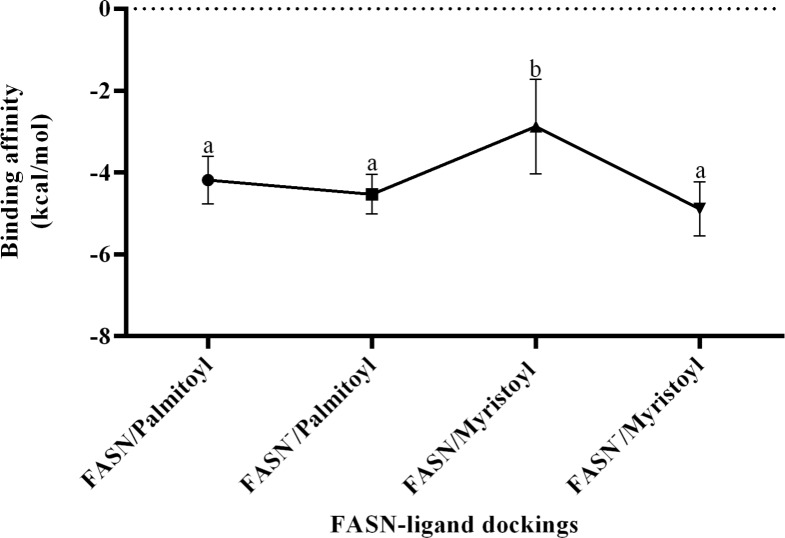
Analysis of variance for FASN/ligand couplings. ^a,b^ Different letters indicate a statistical difference ( p<0.05 ).

## 4 Discussion

### 4.1 Fatty acid profile of intramuscular fat

It has been reported that the SNP FASN g.17924A > G, identified by Maharani et al. (2012), could significantly impact the fatty acid profile of meat by altering the specific activity of the thioesterase domain of FASN on the 14-carbon chain (C-14 acyl-ACP), thereby modifying the ratio between myristic and palmitic acids (Abe et al., 2009; Bhuiyan et al., 2009). In this context, Maharani et al. (2012) associated the g.17924A > G SNP with a higher content of C14:0 in animals with the AA genotype. Similarly, Zhang et al. (2008) reported that Angus cattle with the AA genotype also exhibited increased levels of C14:0, which aligns with the findings of this study. Additionally, Inostroza et al. (2013) found that the GG genotype in Angus steers was associated with higher contents of C16:0, C18:0, and C18:1 fatty acids.

According to reports by Maharani et al. (2012) and Zhang et al. (2008), this study shows that the GG genotype exhibits a lower content of SFAs compared to the AA and AG genotypes in the *Longissimus* muscle profile, as well as an increase in the proportion of myristic and palmitic acids. Additionally, SFAs were predominant in the *Longissimus* muscle, though oleic acid was present in the highest percentage.

In a separate study involving Korean cattle (Hanwoo), the AA genotype was associated with higher levels of C14:0, C16:0, and C18:0 and lower levels of C18:1 in the *Longissimus* muscle (Oh et al., 2012). Recently, another study documented a greater concentration of fatty acids corresponding to C19:1 n-9 and C24:1 n-9 in samples of the *Longissimus* muscle from the AA genotype (Pećina et al., 2023).

Unfortunately, there is a limited number of studies investigating the effects of the FASN g.17924A > G SNP on meat from Holstein Friesian cattle, as most research has focused on milk production (Alim et al., 2014; Ciecierska et al., 2013; Nafikov et al., 2009). Thus, this study provides new information regarding the effect of this mutation in this breed.

### 4.2 In silico evaluation of FASN's interaction with its ligands, myristoyl and palmitoyl

Based on the interaction analysis of the FASN and FASN^−^ models with their ligands (Fig. 2), it is proposed that the affinity of palmitoyl for both models remains standard and is not altered by the amino acid change. In contrast, the normal affinity of FASN for myristoyl ( - 2.878 kcal mol^−1^) is significantly lower than the affinity of both models for palmitoyl ( - 4.186 and - 4.533 kcal mol^−1^ for FASN and FASN^−^, respectively). The alteration induced by the amino acid change is suggested to increase the affinity of FASN^−^ for myristoyl ( - 4.889 kcal mol^−1^) to match that observed for palmitoyl.

This phenomenon can be explained by the fact that the wildtype thioesterase domain is more likely to bind to palmitoyl than to myristoyl, despite the amino acid change. As noted by Chakravarty et al. (2004), the thioesterase domain contains a groove that preferentially binds the 16-carbon fatty acid, indicating that this domain exhibits minimal activity towards chains shorter than 14-carbon atoms in mammals.

According to the simulation, achieving an affinity and interaction level of approximately - 4.186 kcal mol^−1^ for the 16-carbon fatty acid with the thioesterase domain is crucial for the removal of palmitoyl from the ACP and its subsequent release into the cytosol. Consequently, the lower affinity of the wildtype thioesterase domain for the 14-carbon fatty acid is considered normal. This indicates that, with lower affinity, the enzyme releases the 14-carbon fatty acid more readily. In contrast, a higher affinity of the mutated domain for the 14-carbon chain prevents its release, leading to its continued elongation to 16-carbons and, as a result, a lower proportion of myristic acid in the fatty acid profile.

It should be noted that these simulations were based on the monomeric structure of FASN, which may differ in its dimeric form. Despite the increased affinity between the catalytic site of the FASN–thioesterase domain and myristoyl, there is a more distant binding between the FASN subunits in the dimeric form as well as reduced enzymatic activity due to the amino acid change. This may affect the interaction between the ACP of one subunit and the catalytic domains of the adjacent subunit (Joshi et al., 2003). Joshi et al. (2003) reported that any structural alteration in the enzyme results in decreased activity. Therefore, reduced enzyme activity may be more significant than the change in the thioesterase domain's affinity for the fatty acid being formed.

## 5 Conclusion

Despite the observed increase in affinity between FASN and myristoyl, which could potentially enhance the release of myristic acid, changes in enzyme structure are likely more significant in affecting the fatty acid profile of intramuscular fat. However, since this simulation was conducted on the catalytic site of the thioesterase domain, it is anticipated that simulations performed on the entire dimeric form for each genotype may yield different results.

## Data Availability

Data can be requested directly from the corresponding author.

## References

[bib1.bib1] Abe T, Saburi J, Hasebe H, Nakagawa T, Misumi S, Nade T, Nakajima H, Shoji N, Kobayashi M, Kobayashi E (2009). Novel mutations of the FASN gene and their effect on fatty acid composition in japanese black beef. Biochem Genet.

[bib1.bib2] Alim MA, Wang P, Wu XP, Li C, Cui XG, Zhang SL, Zhang Q, Zhang Y, Sun DX (2014). Effect of FASN gene on milk yield and milk composition in the Chinese Holstein dairy population. Anim Genet.

[bib1.bib3] Arnaudova M, Brunner TA, Götze F (2022). Examination of students' willingness to change behaviour regarding meat consumption. Meat Sci.

[bib1.bib4] Bhuiyan MSA, Yu SL, Jeon JT, Yoon D, Cho YM, Park EW, Kim NK, Kim KS, Lee JH (2009). DNA polymorphisms in SREBF1 and FASN genes affect fatty acid composition in Korean cattle (Hanwoo). Asian Australas J Anim Sci.

[bib1.bib5] Cancino-Baier D, Muñoz E, Quiñones J, Beltrán JF, Fuentes F, Farías J, Lorenzo JM, Diaz R, Inostroza K, Ferraz JBS (2021). A Non-Synonymous Single Nucleotide Polymorphism in Gene Alters FASN Enzyme Activity in Subcutaneous and Intramuscular Adipose Tissue in Holstein Friesian Steers. Ann Anim Sci.

[bib1.bib6] Chakravarty B, Gu Z, Chirala SS, Wakil SJ, Quiocho FA (2004). Human fatty acid synthase: Structure and substrate selectivity of the thioesterase domain. P Natl Acad Sci USA.

[bib1.bib7] Ciecierska D, Frost A, Grzesiak W, Proskura WS, Dybus A, Olszewski A (2013). The influence of fatty acid synthase polymorphism on milk production traits in Polish Holstein-Friesian cattle. J Anim Plant Sci.

[bib1.bib8] Dagevos H, Verbeke W (2022). Meat consumption and flexitarianism in the Low Countries. Meat Sci.

[bib1.bib9] Dallakyan S, Olson AJ (2015). Small-molecule library screening by docking with PyRx. Methods in Molecular Biology.

[bib1.bib10] De Smet S, Vossen E (2016). Meat: The balance between nutrition and health. A review. Meat Sci.

[bib1.bib11] Folch J, Lees M, Stanley GHS (1957). A simple method for the isolation and purification of total lipids from animal tissues. J Biol Chem.

[bib1.bib12] Font-i-Furnols M, Guerrero L (2022). Spanish perspective on meat consumption and consumer attitudes. Meat Sci.

[bib1.bib13] Graça J, Godinho CA, Truninger M (2019). Reducing meat consumption and following plant-based diets: Current evidence and future directions to inform integrated transitions. Trends Food Sci Technol.

[bib1.bib14] Heo L, Park H, Seok C (2013). GalaxyRefine: Protein structure refinement driven by side-chain repacking. Nucleic Acids Res.

[bib1.bib15] Hu M, Wu P, Guo A, Liu L (2023). Myristic acid regulates triglyceride production in bovine mammary epithelial cells through the ubiquitination pathway. Agriculture.

[bib1.bib16] Inostroza K, Larama G, Sepúlveda N (2012). Fatty Acid Composition (MUFA and CLA) in Bovine Muscle Tissue Related with the Presence of the g. 878TC SCD Gene Polymorphism. Int J Morphol.

[bib1.bib17] Inostroza K, Larama G, Sepúlveda N (2013). Polimorfismo G.17924a
>
G en el gen FASN y su relación con la composición de ácidos grasos (MUFA Y CLA) en la carne de novillos Aberdeen Angus. Revista Científica.

[bib1.bib18] Joshi AK, Rangan VS, Witkowski A, Smith S (2003). Engineering of an active animal fatty acid synthase dimer with only one competent subunit. Chem Biol.

[bib1.bib19] Kelley LA, Mezulis S, Yates CM, Wass MN, Sternberg MJE (2015). The Phyre2 web portal for protein modeling, prediction and analysis. Nat Protoc.

[bib1.bib20] Knaapila A, Michel F, Jouppila K, Sontag-Strohm T, Piironen V (2022). Millennials' Consumption of and Attitudes toward Meat and Plant-Based Meat Alternatives by Consumer Segment in Finland. Foods.

[bib1.bib21] Leroy F, Abraini F, Beal T, Dominguez-Salas P, Gregorini P, Manzano P, Rowntree J, van Vliet S (2022). Animal board invited review: Animal source foods in healthy, sustainable, and ethical diets – An argument against drastic limitation of livestock in the food system. Animal.

[bib1.bib22] López-Pedrouso M, Lorenzo JM, Gullón B, Campagnol PCB, Franco D (2021). Novel strategy for developing healthy meat products replacing saturated fat with oleogels. Curr Opin Food Sci.

[bib1.bib23] Maharani D, Jung Y, Jung WY, Jo C, Ryoo SH, Lee SH, Yeon SH, Lee JH (2012). Association of five candidate genes with fatty acid composition in Korean cattle. Mol Biol Rep.

[bib1.bib24] Mattick JS, Nickless J, Mizugaki M, Yang CY, Uchiyama S, Wakil SJ (1983). The architecture of the animal fatty acid synthetase. II. Separation of the core and thioesterase functions and determination of the N-C orientation of the subunit. J Biol Chem.

[bib1.bib25] Nafikov R, Schoonmaker J, Reecy JM, Spurlock DM, Beitz DC, Koehler KJ, Minick-Bormann J (2009). Effects of A17924G Genotypes Associated with Thioesterase Domain of Fatty Acid Synthase and K232A Genotypes of Diacylglycerol Acyltransferase-1 on Milk Fatty Acid Composition in Holstein Dairy Cows. Animal Industry Report.

[bib1.bib26] Oh D, Lee Y, La B, Yeo J, Chung E, Kim Y, Lee C (2012). Fatty acid composition of beef is associated with exonic nucleotide variants of the gene encoding FASN. Mol Biol Rep.

[bib1.bib27] Pazirandeh M, Chirala SS, Huang WY, Wakil SJ (1989). Characterization of recombinant thioesterase and acyl carrier protein domains of chicken fatty acid synthase expressed in Escherichia coli. J Biol Chem.

[bib1.bib28] Pećina M, Konjačić M, Ugarković NK, Ivanković A (2023). Effect of FASN, SCD, and GH Genes on Carcass Fatness and Fatty Acid Composition of Intramuscular Lipids in F1 Holstein 
×
 Beef Breeds. Agriculture.

[bib1.bib29] Pence HE, Williams A (2010). ChemSpider: An Online Chemical Information Resource. J Chem Educ.

[bib1.bib30] Realini CE, Ares G, Antúnez L, Brito G, Luzardo S, del Campo M, Saunders C, Farouk MM, Montossi FM (2022). Meat insights: Uruguayan consumers' mental associations and motives underlying consumption changes. Meat Sci.

[bib1.bib31] Teixeira A, Rodrigues S (2021). Consumer perceptions towards healthier meat products. Curr Opin Food Sci.

[bib1.bib32] (2018). UniProt: a worldwide hub of protein knowledge. Nucleic Acids Res.

[bib1.bib33] Trott O, Olson AJ (2009). AutoDock Vina: Improving the Speed and Accuracy of Docking with a New Scoring Function, Efficient Optimization, and Multithreading. J Comput Chem.

[bib1.bib34] Wang M, Ma H, Song Q, Zhou T, Hu Y, Heianza Y, Manson JAE, Qi L (2022). Red meat consumption and all-cause and cardiovascular mortality: results from the UK Biobank study. Eur J Nutr.

[bib1.bib35] Waterhouse A, Bertoni M, Bienert S, Studer G, Tauriello G, Gumienny R, Heer FT, De Beer TAP, Rempfer C, Bordoli L, Lepore R, Schwede T (2018). SWISS-MODEL: Homology modelling of protein structures and complexes. Nucleic Acids Res.

[bib1.bib36] Whitton C, Bogueva D, Marinova D, Phillips CJC (2021). Are we approaching peak meat consumption? Analysis of meat consumption from 2000 to 2019 in 35 countries and its relationship to gross domestic product. Animals.

[bib1.bib37] Xie Y, Ma Y, Cai L, Jiang S, Li C (2022). Reconsidering Meat Intake and Human Health: A Review of Current Research. Mol Nutr Food Res.

[bib1.bib38] Zhang S, Knight TJ, Reecy JM, Beitz DC (2008). DNA polymorphisms in bovine fatty acid synthase are associated with beef fatty acid composition. Anim Genet.

[bib1.bib39] Zhang S, Liu T, Brown MA, Wu JP (2015). Comparison of longissimus dorsi fatty acids profiles in Gansu Black Yak and Chinese Yellow Cattle Steers and Heifers. Korean J Food Sci An.

